# Editorial: Early Events During Host Cell-Pathogen Interaction

**DOI:** 10.3389/fcimb.2021.680557

**Published:** 2021-05-19

**Authors:** Patrícia S. T. Veras, Albert Descoteaux, Maria Isabel Colombo, Juliana P. B. de Menezes

**Affiliations:** ^1^ Laboratory of Parasite-Host Interaction and Epidemiology, Gonçalo Moniz Institute, Salvador, Brazil; ^2^ Centre Armand-Frappier Santé Biotechnologie, Institut National de la Recherche Scientifique (INRS), Quebec, QC, Canada; ^3^ Instituto de Histología y Embriología de Mendoza (IHEM), Consejo Nacional de Investigaciones Científicas y Técnicas (CONICET)-Universidad Nacional de Cuyo, Mendoza, Argentina

**Keywords:** early immune response, microoorganism, disease control, disease progression, immunotherapeutic interventions

## Introduction

The outcome of diseases caused by microbial pathogens (Torgerson et al., 2015) depends on the nature of the pathogen and initial host immune response (Aderem and Ulevitch, 2000; Liu and Uzonna, 2012). This Research Topic “Early Events During Host Cell-Pathogen Interaction” includes 1 review and 1 minireview, and 8 original research articles in the investigation of early events of host immune response against microbial infection.

*Toxoplasma gondii*, *Plasmodium* spp., *Leishmania* spp., and helminths, activate the interleukin-33 (IL-33) pathway, known as the ‘alarmin’ route (Tonacci et al., 2019). Nathan et al. revise the crucial role played by the IL-33 axis in host immune response to these parasites by priming the immune system toward a strong T helper type-2 (Th2) response that subsequently promotes pathogen clearance. Alternatively, IL-33 can also participate in infection exacerbation. Additionally, these authors provide insight into novel immunotherapeutic interventions by modulating early host immune response using antibodies against IL-33 pathway elements. The essential role of autoantibodies in malaria continues to be debated (Hogh et al., 1994; Guiyedi et al., 2007). Another review published in this Research Topic by Mourão et al. summarizes recent findings regarding autoimmune response in malaria. Mainly, the authors discuss controversies surrounding autoantibody response that contribute to disease pathogenesis or protection.

Among food-borne diseases, amebiasis, caused by the protozoan *Entamoeba histolytica*, affects approximately 100 million people worldwide (Haque et al., 2003; Lozano et al., 2012). The parasite life cycle involves inter-conversion between trophozoite, that colonizes the mammalian gut, and cyst, which allows disease transmission through the ingestion of contaminated food or water (Haque et al., 2003). The molecular mechanisms governing parasite life cycle remain poorly studied, and cues triggering stage conversion remain unelucidated (Aguilar-Diaz et al., 2010). Singh’s group previously identified a novel *Entamoeba* transcription factor, Encystation Regulatory Motif-Binding Protein (ERM-BP), which contributes to encystation (Manna et al., 2018). Manna et al. further characterized ERM-BP by evaluating its DNA binding functions and nicotinamidase domains *in vivo.* The overexpression of ERM-BP mutants revealed that this protein is an early regulator of development and heat shock response during *Entamoeba* infection. *Clonorchis sinensis* causes another zoonotic food-borne parasitic disease that can infect mammals *via* the ingestion of raw or undercooked fresh fish and shrimp, eventually resulting in host death (Tang et al., 2016). Yan et al. demonstrated that TLR4 might be involved in *C. sinensis* infection in a resistant mouse strain, C57BL/10. The absence of TLR4 in *C. sinensis*-infected mice led to severe inflammation in the liver, bile duct proliferation, and biliary and hepatocellular injury. TLR4def mice immune response exhibited M2-like macrophages, a robust Th2 and a reduced Th1 responses.

Intracellular protozoan parasites infect millions of people worldwide. These parasites can cause severe forms of diseases that may result in death. Among vector-borne diseases, Chagas disease, caused by *Trypanosoma cruzi*, and leishmaniasis by *Leishmania* spp. are considered the most important due to severity and global distribution (Tang et al., 2016; OMS. Organización Mundial de la Salud, 2019). *T. cruzi* chronicity and persistence depend on parasitic capacity to escape host control mechanisms (OMS, 2020). Gutierrez et al. compared the effect of parasite infection on immune modulation by bone marrow-derived dendritic cells (DCs) and epithelial DCs *in vitro*. These authors found that DC can facilitate infection progression depending on parasite growth phase and DC maturation. Following *T. cruzi* invasion, the formation and maturation of parasitophorous vacuoles (TcPV) occurs. Host Rab GTPases are proteins that regulate intracellular vesicular trafficking. Salassa et al. found that endocytic Rabs, including Rab22a, Rab5, and Rab21, are selectively recruited to TcPV at early times of infection, followed by Rab7 and Rab39a at later stages. However, recycling and secretory Rabs are not recruited to the TcPV membrane. The contribution of these proteins to successful *T. cruzi* infection was demonstrated using cells transfected with mutated endocytic Rab genes.

Although dogs are considered the main reservoir of visceral leishmaniasis caused by *L. infantum*, most knowledge regarding host-parasite interaction stems from murine models (Cestari et al., 2012; Teixeira-Neto et al., 2014). Nadaes et al. evaluated the potential of DH82, a canine macrophage cell line, response to *Leishmania* infection. A comparison of the responses of DH82 and RAW 264.7 mouse macrophages against *L. infantum* promastigotes revealed that both cell lines similarly supported parasite replication/survival. However, notable differences were found in cytokine production, arginase activity and the release of nitric oxide, providing a model to elucidate several aspects of host cell immune response.

Lipophosphoglycan (LPG) is an abundant *Leishmania* promastigote surface molecule that contribute to parasite phagocytosis and survival within host cells (Mosser and Rosenthal, 1993; Descoteaux and Turco, 1999). When duplication of the *LPG2* gene in the *L. infantum* genome impeded gene targeting by conventional homologous recombination, Jesus-Santos et al. used CRISPR/Cas9 technology to generate *Δlpg2* mutants that displayed impaired capacity to infect neutrophils, reinforcing the role of LPG as a virulence factor.

The apicomplexan parasite *T. gondii* causes worldwide foodborne infections in mammals following the consumption of contaminated water or raw fruits or vegetables (Guha-Niyogi et al., 2001; Flegr et al., 2014). *T. gondii* oocysts can cause infections in mice in the absence of digestive factors, differentiating into replicative tachyzoites following phagocytosis by macrophages (Shapiro et al., 2019). Ndao et al. characterized the dynamics of oocyst phagocytosis at the single-cell level. The improved development of tachyzoites in macrophages challenged with free sporocysts or sporozoites compared to whole oocysts suggests that oocyst wall disruption hampers sporozoite excystation in macrophages. Once internalized, *T. gondii* reprograms host gene expression, including the up-regulation of mTOR-dependent host mRNA translation, resulting in the establishment of infection (Al-Bajalan et al., 2017). In addition to the mTOR-4E-BP1/2 axis, MAPK-interacting kinases 1 and 2 (MNK1/2) control the activity of eIF4E, a mRNA cap-binding protein (Leroux et al., 2018). Leroux et al. showed that *T. gondii* inhibits the phosphorylation of axis downstream targets, MNK1/2 and eIF4E, in mouse macrophages. These authors also demonstrated higher *T. gondii* replication in macrophages mutated at the eIF4E phosphorylation residue *vs.* WT cells. Of note, mutant mice were more susceptible to acute toxoplasmosis and showed exacerbated levels of IFNγ. In all, these data suggest that the MNK1/2-eIF4E axis is required to control *T. gondii* infection, and that its inactivation can be exploited by parasites to promote their survival.

In sum, this Research Topic gives insight into the complexity of host early interactions with various microorganisms, providing information about recent advances on the initial immune response that triggers mechanisms, which result in diseases’ control or progression and identifying targets for future interventions on parasitic infections.

## Author Contributions

PV wrote the first draft and AD, MC and JM edited and commented on the draft. All authors contributed to the article and approved the submitted version.

## Funding

PV holds a grant from CNPq for productivity in research (307832/2015-5).

## Conflict of Interest

The authors declare that the research was conducted in the absence of any commercial or financial relationships that could be construed as a potential conflict of interest.
